# Vertical Artifacts in Lung Ultrasonography: Some Common Clinician Questions and the Related Engineer Answers

**DOI:** 10.3390/diagnostics12010215

**Published:** 2022-01-16

**Authors:** Marcello Demi, Natalia Buda, Gino Soldati

**Affiliations:** 1 Department of Bioengineering, Fondazione Toscana Gabriele Monasterio, 56126 Pisa, Italy; demi@ftgm.it; 2 Department of Internal Medicine, Connective Tissue Diseases and Geriatrics, Medical University of Gdansk, Ul. Smoluchowskiego 17, 80-952 Gdansk, Poland; 3 Ippocrate Medical Center, 55032 Lucca, Italy; soldatigino@yahoo.it

**Keywords:** lung ultrasonography LUS, B lines, vertical artifacts, sonomorphology of artifacts, cardiac edema, pulmonary fibrosis, interstitial pneumonia

## Abstract

Introduction: Vertical artifacts, including B lines, are frequently seen in a variety of lung diseases. Their sonomorphology varies in length, width, shape, and internal reverberations. The reason for this diversity is still unknown and is the cause of discussion between clinicians and ultrasound physics engineers. Aim: The aim of this work is to sum up the most common clinician observations and provide an explanation to each of them derived from ultrasound physics. Materials and Methods: Based on clinical and engineering experiences as well as data collected from relevant literature, the sonomorphology of vertical artifacts was analyzed. Thirteen questions and answers were prepared on the common sonomorphology of vertical artifacts, current nomenclature, and clinical observations. Conclusions: From a clinical standpoint, the analysis of vertical artifacts is very important and requires that further clinical studies be conducted in cooperation with engineers who specialize in physics.

## 1. Introduction

The utrasound assessment of the lungs involves the analysis of vertical artifacts and consolidations. B-line artifacts are the most frequently described pathology-related vertical artifacts. The definition of a B-line artifact was established many years ago and was based on the first clinical studies which had been conducted on patients with cardiac insufficiency and pulmonary edema. B-line artifacts also occur in many other diseases affecting the interstitial space and alveoli [1,2]. However, the origin of B lines and other vertical artifacts has still not been thoroughly explained. Varied etiology and pathophysiology of diseases that involve the interstitial space and alveoli result in the presence of vertical artifacts, termed B lines, which can be viewed on the display monitor of the ultrasound machine. Recently, studies devoted to varied sonomorphologies of vertical artifacts have appeared [3]. This publication presents the discussion between two clinicians and a physics engineer as regards the differences in the sonomorphology of vertical artifacts and the impact of physical factors and ultrasound machine settings on vertical artifacts.

## 2. Material and Methods

Some of the terms that will be used in this paper are explained below:

Aerated space/area/volume—air component of the lung (the walls of the alveolar sacs are excluded).

Acoustic medium—includes blood and all other pathological/physiological media that form the barriers that confine air and blood to separate lung compartments.

Acoustic channel—a path given by an acoustic medium surrounded by aerated spaces.

Acoustic trap—a volume of acoustic medium surrounded by aerated spaces that is connected to the pleura plane (and consequently to the thoracic wall) through an acoustic channel.

Aperture—the gap between the aerated spaces that gives rise to an acoustic channel.

Acoustic beam—the area within which the acoustic energy, which is transmitted from the probe to the pleura plane, is mostly constrained through the focusing process. It is characterized by a variable lateral size, which depends on the focus position [4].

Based on clinical and engineering experiences as well as data collected from relevant literature, the sonomorphology of vertical artifacts was analyzed. This work presents examples of clinical observations regarding the presence of vertical artifacts that are manifested in specific clinical conditions, e.g., cardiogenic pulmonary edema, interstitial pneumonia, and pulmonary fibrosis secondary to interstitial lung disease. Questions regarding the reasons for the presence of these related vertical artifacts and the potential impact of the ultrasound machine settings are posed to the engineer who specializes in ultrasound physics.

## 3. Discussion between the Clinicians and the Engineer

**Figure 1:**
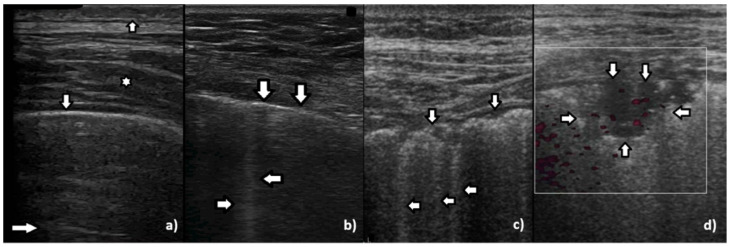
Gradual loss of lung aeration in ultrasound. (**a**) normally aerated lung, (**b**) single vertical artifacts, (**c**) multiple vertical artifacts, and (**d**) subpleural consolidation.

Question 1:

While monitoring the interstitial and alveolar involvement, we observe more and more confluent B lines which can evolve in a so-called white lung artifact which, in turn, can subsequently change to consolidation (see Figure 1) [5,6]. What does the image of B lines and consolidations depend on?

Answer 1:

Isolated B lines are related to isolated acoustic traps and this probably occurs in the first stages of a pathology. When the number of the acoustic traps increases, due to the progression of a pathology, numerous B lines are observed. When their number further increases, they are close to each other and can be even mutually linked. In this case, confluent artifacts logically appear. When the ratio between aerated spaces and interstitial volumes drastically decreases within a lung region, then a lung configuration where small separated aerated spaces are immersed in an interstitial volume is expected. This is probably the lung configuration which is at the basis of the White Lung artifact. Obviously, when the last small aerated spaces also disappear, a clear consolidation naturally emerges.

**Figure 2:**
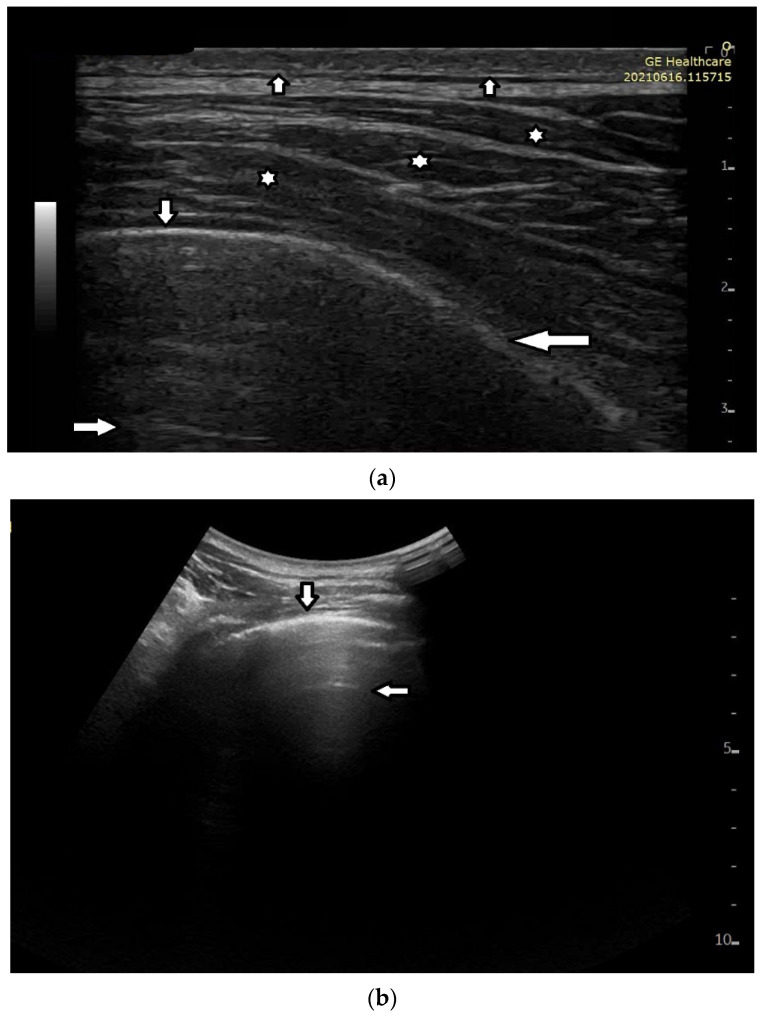
(**a**) Lung ultrasound, linear probe: white stars (*) muscles of the chest wall; down arrow (↓) well-defined pleural line (perpendicular ultrasound beam); left arrow (←) blurred pleural line secondary to the tangential incidence of the ultrasound beam; up arrow (↑) subcutaneous tissue; right arrow (→) A line, horizontal artifact. (**b**) Lung ultrasound, convex probe: down arrow—pleural line, smooth, echoic and regular; left arrow—A-line artifact.

Question 2:

In healthy individuals, an ultrasound examination of the lungs visualizes only the lung surface, termed the pleural line (see Figure 2a). A-line artifacts are visible below the pleural line. These form horizontal lines that appear at regular intervals, equaling the distance between the body surface and the pleural line (see Figure 2b). Under anatomical conditions, it is not possible to visualize lung parenchyma and the interstitium. Only when subpleural areas of the lungs lose their aeration due to lesions can vertical artifacts and/or subpleural consolidations be observed [7]. Why is this so?

Answer 2:

Once an US pulse reaches the pleura plane through the chest wall, it is partially reflected toward the probe and partially transmitted to the channels provided by a specific distribution of the aerated spaces, which characterizes the outer lung surface. In a healthy lung, the size of the interalveolar septa is, and reasonably so, supposed to be comparable to a capillary lumen (less than 10 microns), and an US pulse “sees” this aerated space distribution as a compact air wall. In this case, the acoustic energy is essentially reflected back to the probe. The lung surface is highlighted with a thick white line (the so-called pleural line), and A-line artifacts are visible below the pleural line thanks to the multiple reflections of the acoustic wave between the lung surface and the probe. Replicas of the thoracic wall structures are also often well visible between every pair of A lines thanks to the replica and mirror effects as a consequence of the strong reflection of the lung surface. It is worth noting, however, that the affirmation “The A line artifacts form horizontal lines that appear at regular intervals equaling the distance between the body surface and the pleural line” is not entirely correct. Such an affirmation is correct only if the pleural plane is exactly parallel to the head of the probe [8].

On the other hand, in the presence of thickened interstitial spaces, the pulse energy can be partially trapped by the latter. This can subsequently be reradiated toward the probe after multiple reflections between the separated aerated spaces, giving rise to vertical artifacts, which arise from the pleura line. The imaging parameters play a fundamental role in the formation of the artifacts, and the visibility of a vertical artifact (that is, its brightness, lateral dimension, and length) depends on multiple non-orthogonal factors including the gain, the time gain compensation (TGC), and all the parameters that can be easily set by the operator from the scanner keyboard. The clinical information that can be obtained from lung US images is essentially artifactual information except in the case of consolidation when anatomical information is obtained.

Question 3:

The pleural line is an artifact and, in reality, consists of two opposing layers of serous membranes moving against each other. The apparent movement of the pleural line is termed “lung sliding”. However, when imaging the lungs with a low-frequency probe, e.g., a convex probe or a low-frequency linear probe, it is not possible to differentiate between the two membranes in the pleural line. Why?

Answer 3:

The thick white line which is visible on lung images, which we usually call pleural line, is given by the reflection of the aerated space distribution, which characterizes the outer lung surface. Such a bright white line cannot be related to the reflection of the pleura (an organ which includes the two parietal and visceral pleurae and the pleural cavity), since its acoustical characteristics are similar to those of the thoracic wall and, consequently, it cannot give rise to strong reflections. Moreover, even though a minimal reflection is conceivable at the border between the chest wall and the pleura (i.e., at the parietal pleura), the latter cannot be easily perceived due to the near and much stronger reflection that occurs at the border between the pleura and the outer lung surface (i.e., at the visceral pleura). As a rule of thumb, two different acoustic discontinuities can be separately perceived if the distance between them (in this case, the pleura thickness) is greater than half the pulse length. Let us suppose the pleura thickness be approximately 0.3 mm–0.4 mm. In theory, a short US pulse (provided for example by a linear probe when using a carrier frequency of 6 MHz) can separately highlight the two pleura borders. However, the reflection which occurs at the parietal pleura is minimal with respect to the reflection that occurs at the visceral pleura. I have never studied the problem in depth, but this is probably why the pleura parietal border is not clearly visible (even with a higher frequency).

Question 4:

The head of the probe should be placed over the patient’s body in such a way as to make ultrasound waves perpendicular to the lung’s surface. It should be remembered that the surface of the lung is curved. When the ultrasound beam is perpendicular to the lung surface, the pleural line is well defined, echoic, and smooth under normal conditions. In the case of a tangent ultrasound beam, the pleural line is irregular and simultaneously blurred, which should not be interpreted as a pathological sign, but as a result of an incorrect assessment technique. Can you explain this circumstance better?

Answer 4:

The thickness of the bright white line (the so-called pleura thickness), which highlights the outer lung surface, is related to the pulse length. However, even in the case of a healthy lung, its thickness is theoretically equal to the length of the pulse only if the direction of the acoustic wave propagation is orthogonal to the pleura plane. According to Snell’s law, the reflection angle is equal to the incidence angle, and if the direction of the incidence wave is not orthogonal to the pleura plane, then the probe receives only diffuse energy. In this case, a blurred and thicker “pleura line” should be expected. The problem is evident when using a convex probe since the latter transmits the US pulses (and receives the echoes) in a fan-shaped mode. In this way, on the lateral parts of the fan, the US beam does not intersect the lung surface orthogonally, and the pleura line at the border of the fan is mostly blurred.

Question 5:

We observe B lines in many different clinical conditions, e.g., cardiac edema, interstitial pneumonia, ARDS, pulmonary fibrosis, alveolitis, etc. Each time the interstitial space is involved, B lines are observed [9,10,11]. Which medium could result in visible B lines?

Answer 5:

Whenever the distance among a group of aerated spaces increases, due to the thickening of the barrier that separates them, an acoustic trap develops. An acoustic trap is a volume of non-aerated material and can be a mix of different biological media (water, tissue, blood, exudate, transudate, etc.) with similar acoustic properties. If a reasonably sized acoustic channel, which links the trap to the pleura plane exists, then an acoustic pulse can enter the trap and be gradually reradiated as a vertical artifact. The artifact characteristics are mostly related to the size and shape of the trap and of the linking channel. In my opinion, the impact of the trap medium on the artifact cannot be quantified unless the geometric characteristics of both the trap core and channel are precisely known.

Question 6:

Experts from the World Federation for Ultrasound in Medicine and Biology (WFUMB) differentiate B-line artifacts (BLA) from comet-tail artifacts (CTA) [12]. B-line artifacts arise from the normal pleural line and implicitly result from cardiogenic pulmonary edema. CTA arise from the abnormally changed pleural line, thus they occur in other numerous diseases involving the interstitial space, e.g., pulmonary fibrosis and interstitial pneumonia [13,14,15].

From a clinical standpoint, it seems that such a division is still not ideal. Active lesions in interstitial lung disease, visualized as the presence of “ground glass” in computed tomography, will also be represented as B lines arising from the normal pleural line in the ultrasound image. This happens in acute hypersensitivity pneumonitis, some cases of sarcoidosis, acute interstitial pneumonia, and alveolar hemorrhage [16,17].

We return, then, to physics and the medium of ultrasound wave propagation. It appears that based on the sonomorphology of comet-tail artifacts per se we are unable to differentiate whether the artifact is of a cardiogenic or pulmonary aetiology. Thus, is the transudate secondary to pulmonary edema the reason for the formation of B lines, or is the artifact caused by the inflammatory fluid?

Answer 6:

A vertical artifact can extend from the pleural line to the bottom of the screen, but it can also be shorter; it can appear as a sequence of alternating white and black horizontal bands or as a vertical bright stripe with a constant gray level, and it can be narrow or wide. The spatial distribution of the gray level bands within the vertical artifact can be the expression of a precise periodic function or an aperiodic function. These vertical artifacts are usually named modulated artifacts even when their modulation is a bit confused (when the horizontal bands are not exactly parallel). Obviously, a vertical artifact is also affected by speckle noise and by the superposition of the replica and mirror effects of the chest wall structures; however, this overlapping alone is not sufficient to explain the different structures of the observed B lines. Their structure can even be that of a completely random gray level distribution (confused vertical artifacts).

The visual aspect of a vertical artifact is not strictly correlated to a pathology. The characteristics of an artifact are related to the mutual distribution of aerated and interstitial spaces within which the artifact has been generated. For example: (i) A modulated artifact is probably obtained only when the access channel to the trap core is small with respect to the wave length of the carrier frequency. (ii) A confused artifact is obtained when the channel aperture increases. (iii) A long artifact (either confused or modulated) is obtained when the channel cross section is small with respect to the trap internal surface so that the reradiation of the trapped energy can be slowed down, and so on. From a technical point of view, these configurations of aerated and interstitial spaces, which underlie the vertical artifacts, can arise in different pathologies. Vertical artifacts appear on lung images every time the thickness of the interstitial medium (tissue, blood, and effusion caused by either transudate or exudate), which separates the aerated spaces, increases over a certain threshold. The acoustic properties of transudate and exudate are probably similar and do not allow us to distinguish them on the basis of the US response if these two processes give rise to an identical distribution of aerated and interstitial spaces. What I mean is that you should not try to distinguish transudate from exudate fluids. You should focalize your attention on the potential differences between the distributions of aerated spaces originating from different pathological processes, that is, if there is any obvious difference.

**Figure 3:**
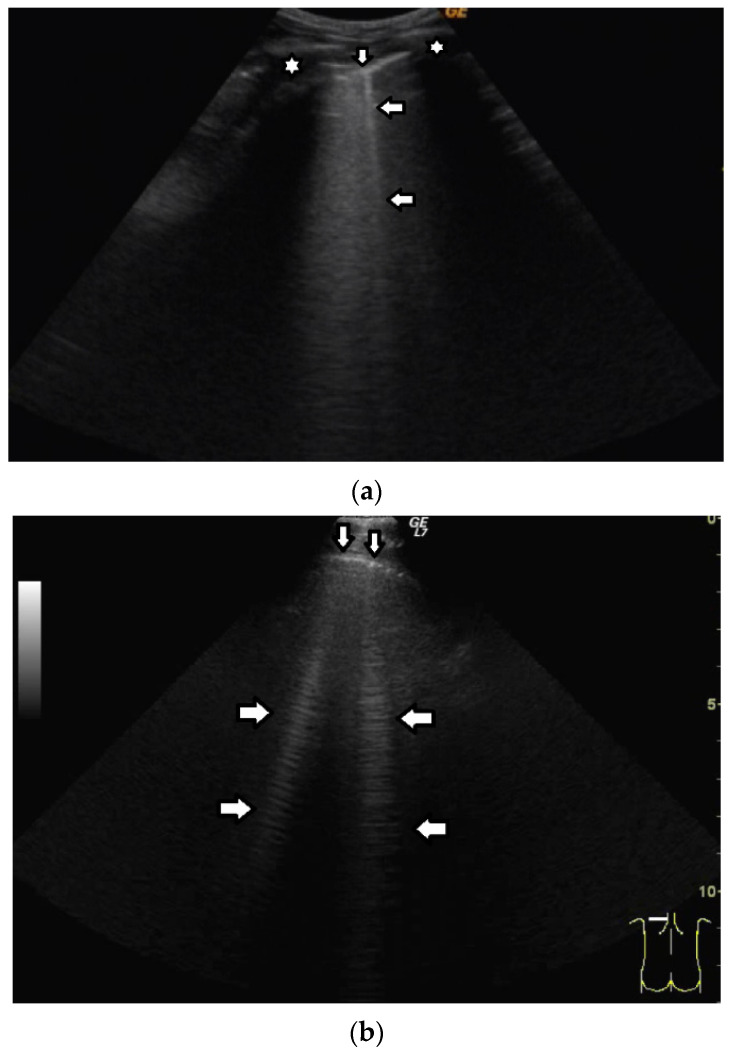
(**a**) Vertical artifact (←) originating from the pleural line (↓), smooth in structure, and ending after a few centimeters (a few centimeters in length). (**b**) Vertical artifacts (←,→) originating from the pleural line (↓), modulated sonomorphology of the artifact, and ending after a few centimeters (a few centimeters in length).

Question 7:

Short vertical artifacts (e.g., the so-called I or Z lines) have never been studied thoroughly. The length of vertical artifacts can vary even in the group of artifacts, which does not reach the bottom of the screen (see Figure 3a,b). One example is the early stage of developing atelectasis (during general anesthesia). What are the most important factors that influence the length of vertical artifacts?

Answer 7:

The length of an artifact, as well as its structure and its brightness, depends on so many factors that it is nearly impossible to list them all [18,19]. Generally speaking, the length of a vertical artifact is given by the time an acoustic trap needs to reradiate the pulse energy, which has been partially trapped therein previously. The length of a vertical artifact not only depends on the geometric characteristic of the acoustic trap but also on the imaging parameters. Therefore, the same acoustic trap may give rise to vertical artifacts with different lengths. The imaging parameters play a fundamental role in the formation of the artifacts and the visibility of a vertical artifact (that is, its brightness, lateral dimension, and length) depends on multiple non-orthogonal factors including the gain, the time gain compensation (TGC), and all the parameters that can be easily set by the operator from the scanner keyboard. Therefore, given the intrinsic variability of the artifacts as a function of multiple nonindependent factors, including the human factor, making an objective diagnosis on the basis of the artifactual information is a difficult task. However, going back to your primary question about the length of the shorter artifacts, I can formulate some hypotheses. First of all, we must answer another question: why are they so short with respect to the artifacts that physicians classify them as “artifacts that extend to the bottom of the screen”? Maybe their linking channel is so small that it allows the transmission of a minimal part of the pulse energy, but in this case, a modulated artifact should appear since this is the peculiarity of a small channel [20]. If a confused artifact is generated, then its minimal length can be related to a wide channel with respect to the core of the trap, which allows a quick release of the trapped energy or a dispersion of the trapped energy through lateral doorways. Attenuation can also theoretically account for a minimal length of the artifact, but in this case, I do not have a valid hypothesis on the nature of the medium.

**Figure 4:**
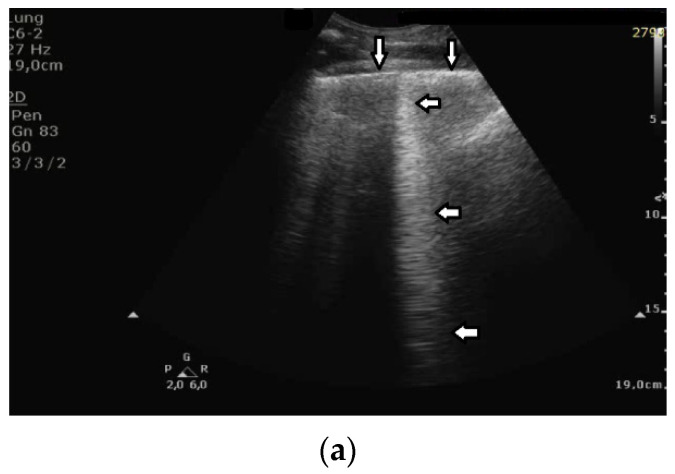

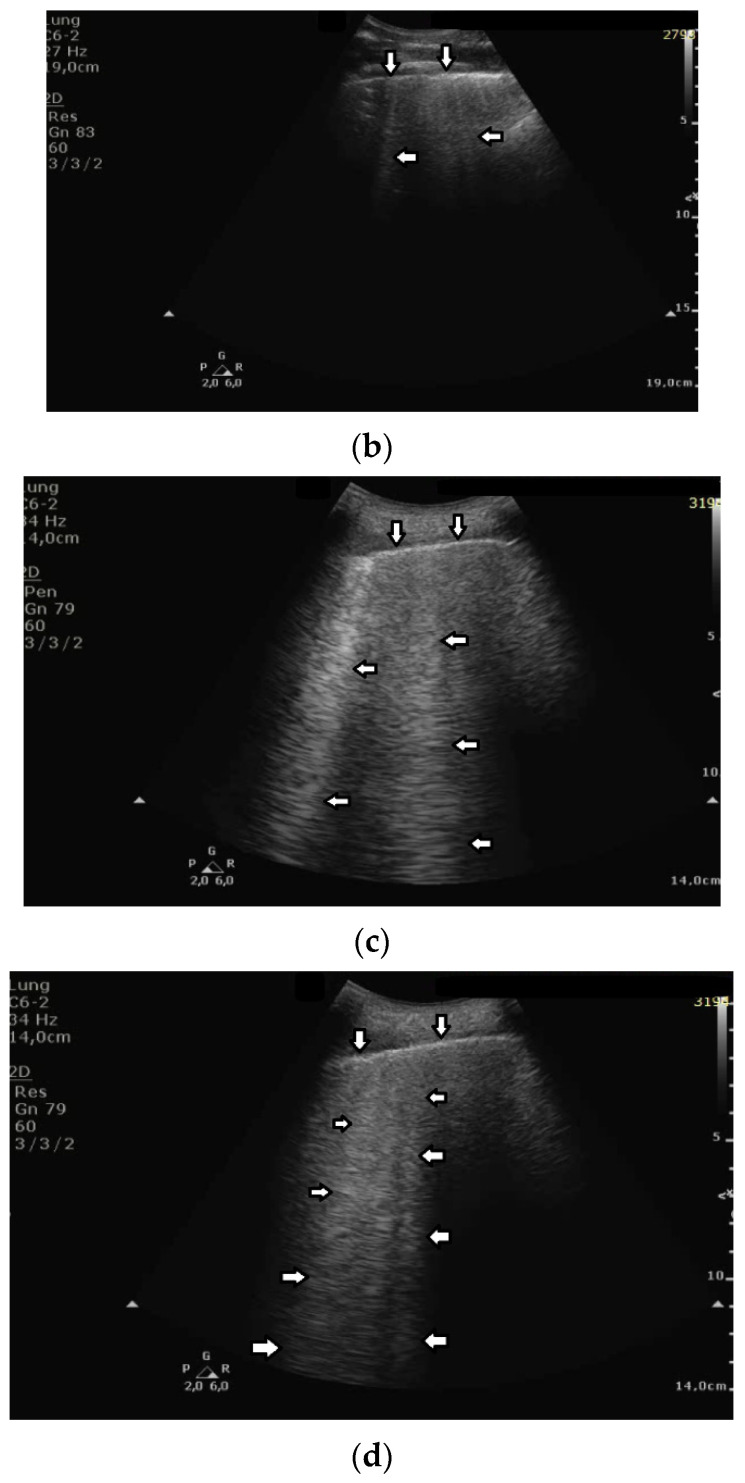
(**a**) Pulmonary fibrosis in the course of interstitial lung disease: down arrows—pleural line; left arrows—B line artifact observed at 2 MHz frequency. (**b**) Pulmonary fibrosis in the course of interstitial lung disease: down arrows—pleural line; left arrows—vertical artifacts observed at 6 MHz frequency. The image was obtained from the same patient and identical assessment site as in Figure 4a. (**c**) Cardiac edema: down arrows—pleural line; left arrows—B-line artifacts observed at 2 MHz frequency. (**d**) Cardiac edema: down arrows—pleural line; left arrows—B-line artifacts observed at 6 MHz frequency. The image was obtained from the same patient and identical assessment site as in Figure 4c.

Question 8:

Based on clinical observations, we know that in pulmonary fibrosis, B lines often reduce their length when higher frequencies are used on a convex probe (from 2 MHz up to 6 MHz) (see Figure 4a,b). In the case of cardiac edema, the length of a B line is often stable, irrespective of the frequency modification (see Figure 4c,d) [3]. Why does the length of B lines sometimes change when varying the pulse central frequency, whereas sometimes the length does not significantly change?

Answer 8:

Look at the entire image, and you will find the answer. When using the 2 MHz frequency, the image (Figure 4a) is brighter everywhere: the thoracic wall is brighter; the pleura line is brighter; the two lateral sides of the image (where there are no artifacts) are brighter, and the artifacts themselves are brighter. In my opinion, the problem is primarily given by the attenuation, which increases when increasing the frequency. In order to compensate such an effect, you should change the TGC. As a rule of thumb, you can consider an attenuation coefficient of 1 db/cm/Mhz. When varying the frequency from 2 MHz to 6 MHz, you are introducing an additional attenuation of 4db per centimeter. From a practical point of view, when using the 6 MHz frequency, the probe receives a signal from the depth of half a centimeter, whose amplitude is less than half the amplitude of the signal it would receive from the same depth if a 2 MHz pulse were used. Moreover, it is worth noting that the ratio between the amplitudes of the two temporal signals s_6_(t) and s_2_(t) (the echoes received by the probe) decreases exponentially when the delay t increases.

The answer is a bit more complex when cardiac edema is considered. In the case of fibrosis, larger acoustic traps and wider linking channels are, in general, expected that reradiate almost the entire power spectrum of the trapped acoustic pulse. In this case, attenuation is probably the main factor that influences the artifact length when varying the frequency. Therefore, the artifact length decreases when increasing the pulse central frequency since attenuation increases when increasing the frequency. On the contrary, in the case of cardiac edema, smaller acoustic traps and narrower linking channels are expected at the early stages of the pathology. Smaller traps can reradiate only a few harmonics, and the overlapping between the pulse spectrum and the spectral signature of the trap affects the artifact length much more than the attenuation factor. In these cases, the trap response to pulses with different frequencies is unpredictable.

**Figure 5:**
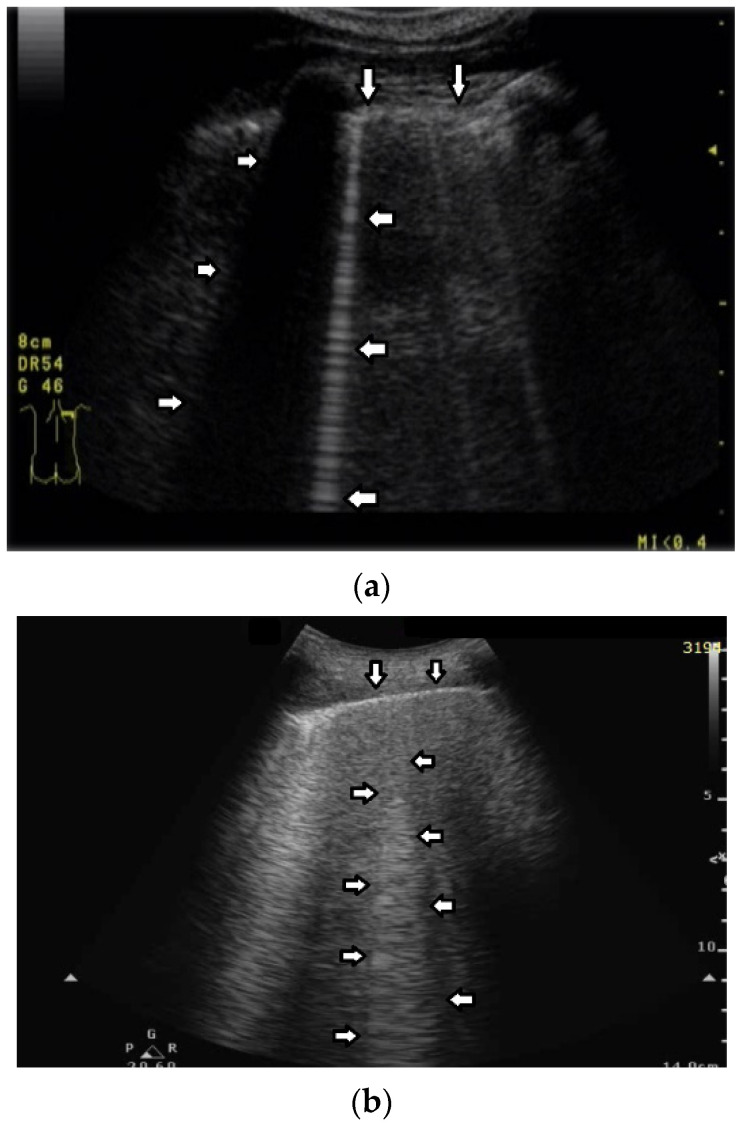

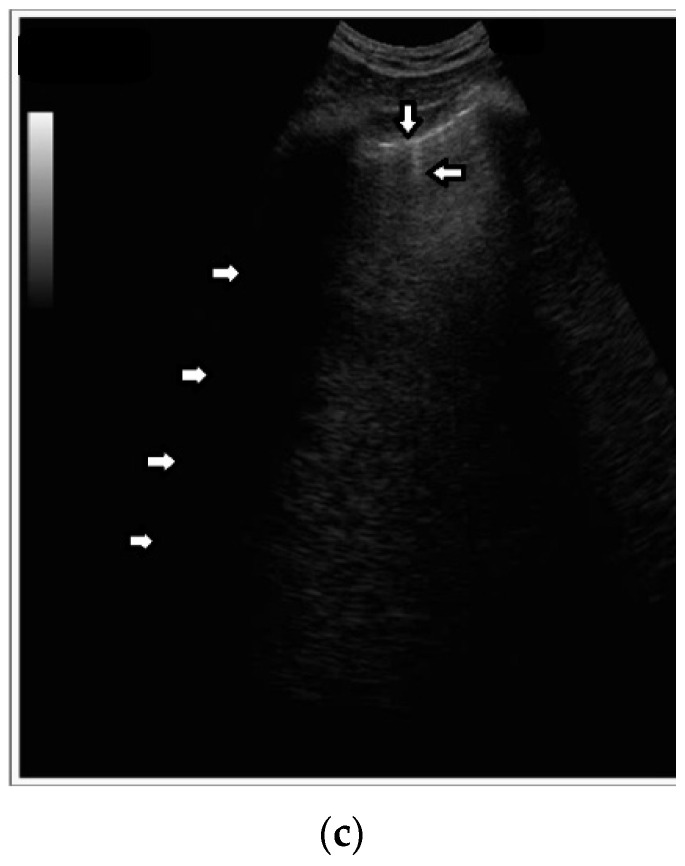
(**a**) Irregular, echoic pleural line (vertical arrows), long vertical artifact (left horizontal arrows), and shadow behind the rib (right horizontal arrows). The vertical artifact is classified as a B line since it originates from the pleural line and reaches the edge of the screen. Multiple small horizontal artifacts (named J artifacts by D. Lichtenstein) are visible inside the B line. (**b**) Smooth, regular, and echoic pleural line (vertical arrows), long vertical artifact, so called B line: narrow at the top and wide at the bottom (left and right horizontal arrows). (**c**) Smooth, regular, and echoic pleural line (vertical arrow), short vertical artifact, so called I line (left horizontal arrow and shadow behind the rib (right horizontal arrows).

Question 9:

Based on clinical observations, we know that vertical artifacts can have different lengths, widths, and shapes (see Figure 5a–c). A modulation of artifacts is also often clearly perceived. Why are they so varied? What does this depend on?

Answer 9:

Many types of acoustic traps exist, and, consequently, many types of vertical artifacts exist. Let us consider an isolated acoustic trap. It has its own volume and geometric shape. It is linked to the pleura plane by means of an acoustic channel (typically an interalveolar interstice), and this channel also has its own shape and size represented by its cross-section and length. Every single difference between two acoustic traps affects the visual aspect of the related artifacts. Moreover, you should never forget that even the imaging parameters (pulse central frequency and bandwidth, focus position, TGC, pulse amplitude, etc.) strongly affect the formation and the final shape of an artifact.

Question 10:

B lines that meet the assumptions of the definition may differ from each other. Why do we observe so many sonomorphologies of B lines when we use a convex probe? B lines can be:

(a) Wide at the edge of the screen.
(b) Narrow throughout the entire length.
(c) Modulated.
(d) Smooth.
(e) Frequency dependent or not.

Answer 10:

Usually, when using a convex probe, the lateral size of a vertical artifact naturally increases far from the pleura plane due to the signal/image processing algorithms that the US system uses to convert RF signals into a two-dimensional image. Moreover, the lateral size of an artifact (provided by a convex probe) at the end of the screen depends on the image depth. A constant narrower artifact is, on the contrary, common when using a linear probe. Modulated artifacts are observed when the cross section of the acoustic channel that links the trap to the pleura plane is small with respect to the wave length of the pulse central frequency. Here, a hypothesis can be formulated: when the link between the trap and the pleura plane is reduced to a small acoustic channel, the trap acts as a point-like source of ultrasound and, consequently, can eliminate the uneven acoustic perturbation of the particles of the medium at the top of the channel [20]. If by “smooth artifact” you mean a vertical artifact with a unique gray level from the beginning to the bottom, this is a particular type of modulation, which is obtained when a trap reradiates a single harmonic. The vertical artifacts are always frequency dependent since this is an intrinsic characteristic of the ultrasound investigation. The variation of the ultrasound response with the frequency may not be always visually perceptible, but such a variation always exists.

Question 11:

The length of vertical artifacts can vary. Consistent with the terminology proposed by Daniel Lichtenstein, B, I, Z, and C lines are differentiated. B, Z, and I lines originate from the pleural line, but have various lengths. Thus far, Z and I lines have not been considered to be clinically significant. C lines originate from the lower edge of subpleural consolidations.

**Figure 6:**
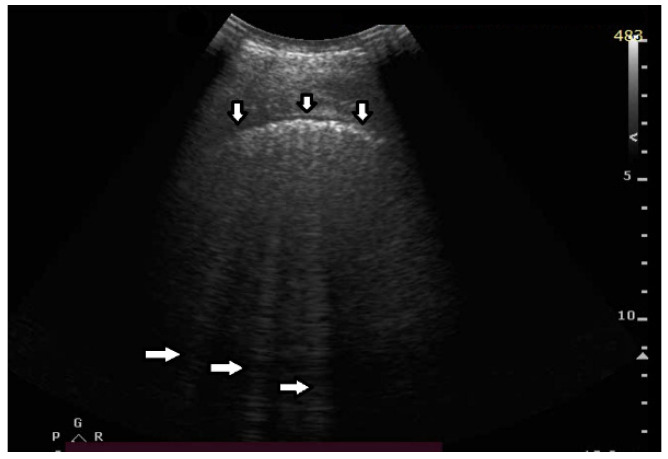
B lines (horizontal arrows) and pleural line (vertical arrows).

The significance of B lines is related to lesions affecting the interstitial space and alveoli (see Figure 6). Most frequently, the presence of B lines is reported in cardiogenic pulmonary edema, interstitial pneumonia, ARDS, and pulmonary fibrosis secondary to interstitial lung disease. Despite copious clinical observations describing the significance of B-line artifacts, we have still not acquired complete knowledge as regards their origin. The definition of a B line that is presently adopted is as follows:

(a) Well defined.
(b) Originating from the pleural line.
(c) Going to the edge of the screen.
(d) Laser like.
(e) Erasing A lines.
(f) Moving with lung sliding.

Do you agree with this definition of B line? Should we differentiate B lines from other vertical artifacts?

Answer 11:

It is difficult to challenge a definition since a definition is a definition “by definition”. What I disagree with is the vagueness of this definition. “Moving with lung sliding” is a specific feature which links the artifact to a precise anatomical position of the outer lung surface where an acoustic trap developed. However, “well defined” is a subjective and vague characteristic. Moreover, the characteristic of “originating from the pleural line” is not properly formulated since in theory such a definition excludes B lines, which do not originate exactly at the pleural line. Due to both the size of the acoustic trap and the length of the linking acoustic channel, a B line often starts a bit lower than the pleural plane. “Going to the edge of the screen” is also an ambiguous characteristic since the edge of the screen is decided by the operator when he/she sets the acquisition depth. Even the characteristic of “Erasing A lines” is not properly formulated since some modulated B lines do not completely erase the A lines. In particular, the lateral size of B lines does not change a lot when a linear probe is used, and A lines mostly remain visible in the presence of a few B lines. Moreover, “laser like” is an intuitive description, but it is not specific. “Lying on the direction of the acoustic beam propagation” should be used since this is the specific characteristic.

Question 12:

For clinicians the meaning of B lines depends on many factors:

(a) Does it meet the definition of a B line artifact?
(b) Sonomorphology of a B line.
(c) Gravity dependence.
(d) Distribution of interstitial lesions.
(e) Coexisting ultrasound signs.
(f) Ultrasound image with a linear probe.
(g) And the most important factor—clinical context.

What is your proposal (from an engineer’s point of view) for the classification of vertical artifacts?

Answer 12:

Why should I classify what I am observing into a limited number of cases? A process of information classification is a delicate process since the risk of losing important information is always present. This is particularly risky in our case since we are dealing with artifactual information, and we do not yet know what is really important and what can be neglected. When using an US probe, a physician actively observes a scene through many video clips. He/she should not observe a single artifact in order to classify it according to a rigid scheme. Different types of artifacts are usually observed even on the same image, and their co-existence is additional information. Moreover, it is also important to analyze how the visual characteristics of the artifactual information change when varying the imaging parameters. Nowadays, the artifactual information should be considered as a unique complete picture, which a physician must interpret on the basis of his/her anatomical knowledge and clinical experience. Our knowledge of the artifactual information is not yet sufficiently mature to be usefully synthetized by means of a classification procedure. In my opinion, a physician should learn to read the artifactual information with the objective of translating it in physioanatomical information. He/she should not give in to the temptation of shortcuts, such as those offered by questionable algorithms derived from AI.

Question 13:

What is the difference between “comet-tail” and “ring-down” artifacts?

Answer 13:

In my opinion, the only difference is in the name. The two names have been proposed in different papers by different authors [21,22] and derive from analogies with similar phenomena suggested by the visual aspect of the examined artifacts. The comet-tail artifact was described as a reverberation effect, i.e., multiple reflections of an acoustic wave between two opposite internal walls of a strong reflector. The ring-down artifact was described as a resonance effect, i.e., when one or more frequency components (multiples of the fundamental frequency) of a transmitted signal are highlighted by a receiving apparatus. However, they are two sides of the same coin. In any case, they refer to trapped (absorbed, transmitted, etc.) energy which is subsequently released in the form of a periodic signal. A vertical artifact is generated by an acoustic trap that gradually re-radiates previously trapped energy, independently of its given name (ring-down, comet-tail, B-line, etc.).

## 4. Conclusions

A comparison between US images obtained on the same patient with different imaging parameters (central frequency, bandwidth, focus, etc.) is needed to make the artifactual information significant. The artifactual information depends on so many factors that it is risky to directly translate the visual inspection of a single US cine-loop into clinical information. A classification of the artifactual information on the basis of its visual characteristics can be misleading since the latter is a relative “measure” and must be analyzed by varying the imaging parameters.

Artifactual information must be evaluated along with other clinical information, such as an interview, physical examination, and laboratory and microbiological test results.

The differentiation of vertical artifacts, the assessment of their sonomorphology, the evaluation of the subpleural area with the employment of a lineal probe, the assessment of coexisting lesions visualized in lung ultrasound images, and the interpretation of the detected lesions in the clinical context are crucial for clinicians in the differential diagnosis of the aetiology of lesions affecting the interstitium of the lungs.

From a clinical standpoint, the analysis of vertical artifacts is very important and requires further clinical studies conducted in cooperation with engineers who specialize in physics. Communication between clinicians and engineers is vital considering the further development of lung ultrasound [23].

## Data Availability

Data sharing not applicable.
